# Linking Personality Traits to Disability Progression in Multiple Sclerosis: A Longitudinal Analysis

**DOI:** 10.1093/arclin/acaf063

**Published:** 2025-07-15

**Authors:** Isabele Jacot de Alcântara, Philippe Voruz, Gilles Allali, Patrice H Lalive, Julie A Péron

**Affiliations:** Clinical and Experimental Neuropsychology Laboratory, Faculty of Psychology, University of Geneva, Geneva, Switzerland; Division of Neurology, Department of Clinical Neurosciences, Geneva University Hospitals, Geneva, Switzerland; Clinical and Experimental Neuropsychology Laboratory, Faculty of Psychology, University of Geneva, Geneva, Switzerland; Division of Neurosurgery, Department of Clinical Neurosciences, Geneva University Hospitals, Geneva, Switzerland; Leenaards Memory Center, Lausanne University Hospital and University of Lausanne, Lausanne, Vaud, Switzerland; Division of Neurology, Department of Clinical Neurosciences, Geneva University Hospitals, Geneva, Switzerland; Faculty of Medicine, University of Geneva, Geneva, Switzerland; Clinical and Experimental Neuropsychology Laboratory, Faculty of Psychology, University of Geneva, Geneva, Switzerland; Division of Neurology, Department of Clinical Neurosciences, Geneva University Hospitals, Geneva, Switzerland

**Keywords:** Multiple sclerosis, personality, disability, generalized estimating equations, individual variability

## Abstract

**Objective:**

Living with a chronic disease often involves facing many personal changes. In multiple sclerosis (MS), these changes can occur in both physical and psychological domains. Interestingly, psychological factors, including personality traits, have been suggested as potential contributors to disability progression in MS. However, longitudinal studies exploring these associations are scarce. The objective of this study is to highlight and understand long-term associations between personality and disability progression in MS using advanced statistical methods that provide a robust approach to analyzing repeated measures.

**Methods:**

Twenty-eight people with a confirmed diagnosis of MS (M_age_ = 47.5 years; 19 women) were included in this longitudinal study. Personality was assessed using the NEO-Personality Inventory-3 and disability with the Expanded Disability Status Scale at two time points (mean interval of 4.2 years). Finally, Generalized Estimating Equations were performed.

**Results:**

Persons who displayed an increase in Neuroticism, notably in its Anxiety facet, as well as a decrease in Extraversion, particularly in its Warmth and Gregariousness facets, showed more disability increase.

**Conclusion:**

These findings underscore the importance of considering personality dynamics in the management of MS and advocate for a more individualized, person-centered approach to care and research.

## INTRODUCTION

Multiple sclerosis (MS) is a chronic demyelinating and inflammatory neurological disease that often occurs at the beginning of adulthood (Jakimovski et al., 2024). Having to cope with the announcement of a chronic and evolutive disease can have many psychological consequences, which could partially explain the high prevalence of mood disorders in persons living with MS (PwMS) (Foley, 2015). The most frequent mood disorders are depressive and anxious symptoms (Magistrale & Nocentini, 2015; Théaudin & Feinstein, 2015) which have both been associated with well-being and quality of life (Hanna & Strober, 2020). Beyond these mood modifications, the continuous adaptation to an evolving disease could also impact psychological characteristics considered relatively stable, such as personality.

Indeed, studies have shown that PwMS report changes in personality following their diagnosis (Weddell & Fisher-Hicks, 2021). These personality changes were primarily associated with psychological variables, notably stress reaction, emotional distress, perceived cognitive impairment, and poorer family functioning. Longitudinal evidence further supports this observation: Fuchs et al. (2018) demonstrated a decrease in the personality traits of Extraversion and Conscientiousness over five years, particularly in PwMS experiencing cognitive decline. While it is difficult to express a clear opinion regarding the causality between cognitive decline and personality changes, this finding suggests that, beyond psychological factors, personality changes could also reflect neuroanatomical modifications. Supporting this idea, studies have linked the personality trait of Conscientiousness with both anatomical and functional brain dynamics in PwMS (Fuchs et al., 2018; Fuchs et al., 2020; Fuchs et al., 2022). Together, these findings suggest that personality changes in MS may be associated with disease progression, and several studies have demonstrated links between personality and functional outcomes in MS (Maggio et al., 2020). For instance, a recent work highlighted associations between Neuroticism, Extraversion, and neurological disability, as evaluated by the Expanded Disability Status Scale (EDSS) (Jacot de Alcantara et al., 2023).

However, most existing studies have been cross-sectional (Jacot de Alcantara et al., 2023; Maggio et al., 2020), leaving longitudinal relationships between personality changes and disability progression underexplored. Moreover, most studies focus on broad personality factors without examining specific facets, which could provide deeper insights into how precise traits might contribute to disability progression. Importantly, no study to date has applied Generalized Estimating Equations (GEE) models, which are particularly well-suited for longitudinal analyses. These models allow for the assessment of temporal relationships within individuals, as opposed to comparing predefined groups based on variables like cognition—a method that risks overlooking key longitudinal dynamics.

### Objective and hypotheses

In this context, the present study aimed to test whether associations between personality and disability could be identified longitudinally. Specifically, we investigated whether changes in personality traits were related to disability progression. Based on prior findings (Jacot de Alcantara et al., 2023), we hypothesized that a decrease in Extraversion and an increase in Neuroticism would be associated with greater disability progression over time. We did not postulate any hypothesis regarding the causality of the associations between personality and disability, as our paradigm could not address this complex question. Moreover, we believe there is a dynamic relationship between these aspects, likely involving bidirectional influences. Better understanding the role of personality change in disability progression may support the early identification of patients at risk for functional decline, and ultimately contribute to more personalized and anticipatory follow-up strategies in clinical care.

## METHODS

### Participants

Forty-seven PwMS responded to a personality questionnaire in 2019. As part of an ongoing study supported by the Swiss Multiple Sclerosis Society, we contacted these persons and proposed a second personality evaluation that was conducted between 2023 and 2024. The second evaluation incorporated a comprehensive neuropsychological assessment, which had not been conducted during the first evaluation. The main eligibility criterion was a confirmed MS diagnosis according to the 2017 McDonald criteria (Thompson et al., 2018). The participants were recruited through the neuroimmunology unit of the Geneva University Hospitals. A total of twenty-eight people completed the two evaluations. As the second evaluation was part of a new project, and not originally planned, reasons for attrition were not formally assessed. However, informal feedback collected during recontact indicated that the main reasons for non-participation were lack of time, limited interest, or reluctance to undergo a cognitive evaluation. Given that written consent was obtained during the neuropsychological evaluation and the personality assessment was conducted online, participants did not have the option to complete only the personality assessment. These changes in the experimental setting might explain the attrition at the second evaluation. The mean interval between the two measures was 4.2 years (*M*_days_ = 1524.7, *SD* = 208.4, *range* = 1,371–2,479).

### Measures

The NEO Personality Inventory-3rd edition ((NEO-PI-3, McCrae et al., 2005) was used to assess personality. This questionnaire is based on the Five Factor Model (FFM) that measures the five major factors of personality (Neuroticism, Extraversion, Openness, Agreeableness, and Conscientiousness). Each of these factors is composed of six specific facets (N: Anxiety, Angry Hostility, Depression, Self-Consciousness, Impulsiveness, Vulnerability; E: Warmth, Gregariousness, Assertiveness, Activity, Excitement Seeking, Positive Emotions; O: Fantasy, Aesthetics, Feelings, Actions, Ideas, Values; A: Trust, Straightforwardness, Altruism, Compliance, Modesty, Tender-Mindedness; C: Competence, Order, Dutifulness, Achievement Striving, Self-Discipline, Deliberation). This questionnaire is based on 240 items, such as “I am not the type to worry”, with a response range from 1 “strongly disagree” to 5 “strongly agree”. Each participant obtains a score for each of the thirty facets as well as the five factors. We then converted the scores into standardized T-scores, accounting for sex differences using the NEO-PI-3 French norms (Rolland, 2016). The combination of these different traits allows for a comprehensive assessment of personality.

The EDSS (Kurtzke, 1983), was used to quantify disability. This scale measures the level of disability caused by MS based on different functional systems that are independent of each other, but which together reflect neurological disorders associated with MS. Levels range from 0 (= no disability) to 10 (= death caused by MS). We extracted the EDSS scores from the medical visit closest to the personality assessment.

The mean delay between personality assessment and EDSS was 3.8 months for the first evaluation (*M*_days_ = 114.23, *SD* = 85.78, *range* = 0–278) and 2.3 months for the second (*M*_days_ = 68.62, *SD* = 70.80, *range* = 6–259).

### Statistical analyses

Analyses were run in Jamovi (Version 2.3), IBM SPSS 27 and RStudio 4.4.1.

First, to examine whether there were any differences between the individuals who accepted the follow-up and those who did not, we conducted mean comparison analysis on demographic and clinical variables, as well as personality factors. Then we performed preliminary analyses to evaluate overall trends in the data. Firstly, we applied a unilateral paired-sample Wilcoxon test to determine whether there was a significant increase in disability between the two time points. Secondly, we assessed potential changes in personality traits over the same period using paired-sample t-tests for normally distributed data and Wilcoxon tests for non-normally distributed data. While these analyses provide insights into group-level changes, they do not account for the correlation of repeated measures within individuals, as the GEE models do. Therefore, the use of GEE enables the detection of associations that may not be apparent in the mean comparisons. Results regarding mean comparisons are reported in the Supplementary Material file to ensure transparency and provide comprehensive details about the findings (see Table S1, Figs S1–S3).

Therefore, GEEs were used to explore the relationships between personality traits and disability progression. These models are particularly suited for longitudinal data as they account for the within-subject correlation structure. Moreover, they are well-suited for psychological research with a small sample (Muth et al., 2016). Initially, models included the five broad personality factors, age, and time since disease onset as predictors, as well as the EDSS as the dependent variable, with time as the within-subject effect. For the selection of model parameters, we relied on the Quasi-likelihood under the Independence Model Criterion (QIC) (Cui & Qian, 2007). If significant effects were identified at the factor level, additional GEE models were conducted to investigate these relationships at the facet level. This approach allows for a more detailed understanding of which facets within broader personality factors are most relevant to disability progression. To account for potential overfitting and multicollinearity due to the high dimensionality of our models, we conducted additional analyses using Penalized GEE (PGEE). This method extends classical GEE and is particularly suited for longitudinal data with many and/or correlated predictors (Wang et al., 2012). Analyses were conducted using the PGEE package (Inan et al., 2025). Finally, to account for multiple testing and ensure the robustness of our findings, we applied False Discovery Rate (FDR) corrections to *p*-values in all analyses, including those conducted at the facet level. This approach allows for a more detailed and statistically reliable understanding of which facets within broader personality factors are most relevant to disability progression.

### Supplementary analyses

In order to provide additional information about the characteristics of the individuals included in this study, we conducted supplementary analyses of their cognitive performances. The neuropsychological data was divided into six domains: memory, attention, executive functions, language, visual perception, and ideomotor praxis. Cognitive performances were categorized into seven levels according to the classification of the Swiss Association of Neuropsychology: *well above average, above average, high average, average, low average, below average, well below average*. For each category, we calculated the cumulative percentage of individuals who obtained at least one standardized score falling into that category across the six cognitive domains.

These results are only presented in the supplementary file (Tables S2 and S3).

## RESULTS

### Socio-demographic and clinical data

At the second time-point, the mean age of the people included in this study was 47.5 years old (*SD* = 9.4) and the time since their first symptom was 15.9 years (*SD* = 9.4). Among the 28 persons, 19 were women. Regarding the clinical form of MS, 26 individuals had a Relapsing–Remitting MS and 2 had a Secondary Progressive MS. As for MS treatment, 26 out of the 28 individuals included in the study were receiving appropriate treatment at the time of the second evaluation. The mean EDSS was 1.9 (*SD* = 1.8) at the first time point and 2.2 (*SD* = 1.7) at the second. The unilateral Wilcoxon test revealed a significant increase in disability between these two time points (*W* = 4, *p* < .007). All demographic information, as well as the mean T score on the five factors of personality, can be found in Table 1.

**Table 1:** Demographic and clinical information, as well as personality T scores on the five factors at the two time points

	Age at T2 (in years)	Time since disease onset at T2 (in years)	EDSS T1	EDSS T2	N T1	N T2	E T1	E T2	O T1	O T2	A T1	A T2	C T1	C T2
Mean (*SD*)	47.5 (9.4)	15.9 (9.4)	1.9 (1.80)	2.2 (1.7)	45.9 (12.8)	45.8 (12.0)	54.4 (9.7)	55.0 (11.0)	60.3 (10.9)	59.8 (9.5)	53.1 (12.1)	53.5 (11.6)	54.1 (9.6)	54.9 (7.9)
Minimum	34	8	0.0	0.0	24	26	22	29	30	41	26	28	27	42
Maximum	65	43	8.0	8.0	80	74	72	75	80	80	75	77	74	68

*Note:* Higher T scores in personality factors indicated a higher probability of having the characteristics associated with the personality trait. T1 = first time point, T2 = second time point, SD = standard deviation, N = Neuroticism, E = Extraversion, O = Openness, A = Agreeableness, C = Conscientiousness

Regarding the characteristics of individuals who accepted the longitudinal follow-up compared to those who did not, the former had higher levels in Openness (*t* = −3.53, *p* = .001). No significant differences were observed in age, time since disease onset, sex, MS type, EDSS, and the other personality factors (see Table S4).

### Longitudinal associations analysis

**Fig. 1:**
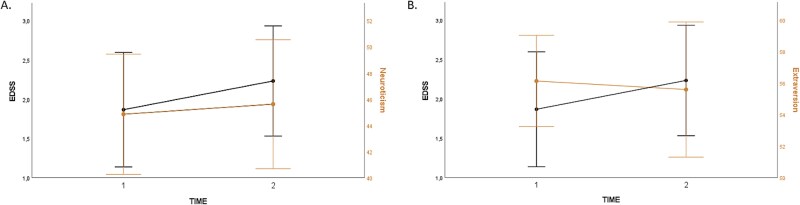
Longitudinal changes in EDSS as well as the personality traits of neuroticism (A) and extraversion (B). *Legends.* Lines in the figures are interpolation lines.

We performed the first GEE model including the variables of age, time since disease onset, and the five factors of personality. Our data included some outliers, which, after careful consideration, were retained in the analysis as they represent expected and meaningful heterogeneity within clinical populations. We used the gamma distribution with a log link function, as our data were not normally distributed and displayed a right-skewed distribution, and an independent working correlation matrix structure, as this enabled a better adjustment of the model (QIC = 27.58). The total sample for GEEs analysis was 25 persons, as the gamma distribution is invalid for data equal to 0 (excluding persons with an EDSS of 0). The variables of age (*β* = 0.03, *p* = .002), time since disease onset, (*β* = .02, *p* = .01), Neuroticism (*β* = 0.02, *p* = .001) and Extraversion (*β* = −0.03, *p* < .001) had significant coefficients after FDR correction. Openness, Agreeableness, and Conscientiousness had non-significant coefficients (*β* = −0.003, *p* = .68; *β* = −0.002, *p* = .74; *β* = 0.011, *p* = .14). Changes in EDSS, Neuroticism, and Extraversion are represented in Fig.  1.

In order to explore the relationships between Neuroticism, Extraversion, and disability more precisely, we performed two additional GEEs including personality data at the facet-level.

The GEE model for Neuroticism included the variables of age, time since disease onset, as well as the six facets of Neuroticism (i.e., Anxiety, Angry Hostility, Depression, Self-Consciousness, Impulsiveness, and Vulnerability). The gamma distribution with log link function and an independent working correlation matrix structure enabled a better adjustment of the model (QIC = 29.83). The variable of age (*β* = 0.029, *p* = .02), time since disease onset (*β* = 0.029, *p* = .02) and the Anxiety facet (*β* = 0.023, *p* = .002) had significant coefficients. The facets of Angry Hostility, Depression, Self-Consciousness, Impulsiveness, and Vulnerability had non-significant coefficients (*β* = −0.005, *p* = .51; *β* = −0.015, *p* = .20; *β* = 0.012, *p* = .20; *β* = 0.008, *p* = .35; *β* = 0.006, *p* = .27).

The GEE model for Extraversion included the variables of age, time since disease onset, and the six facets of Extraversion (i.e., Warmth, Gregariousness, Assertiveness, Activity, Excitement Seeking, and Positive Emotions). Once again, we used the gamma distribution with log link function and an independent working correlation matrix structure, as it provided the best adjustment (QIC = 26.31). The variables of age (*β* = 0.021, *p* = .002), time since disease onset (*β* = 0.025, *p* = .003), the facets of Warmth (*β* = −0.026, *p* < .001) and Gregariousness (*β* = −0.009, *p* = .02) had significant coefficients. The facets of Assertiveness, Activity, Excitement Seeking, and Positive Emotions had non-significant coefficients (*β* = −0.011, *p* = .07; *β* = 0.001, *p* = .91; *β* = −0.009, *p* = .07; *β* = 0.004, *p* = .67).

**Fig. 2:**
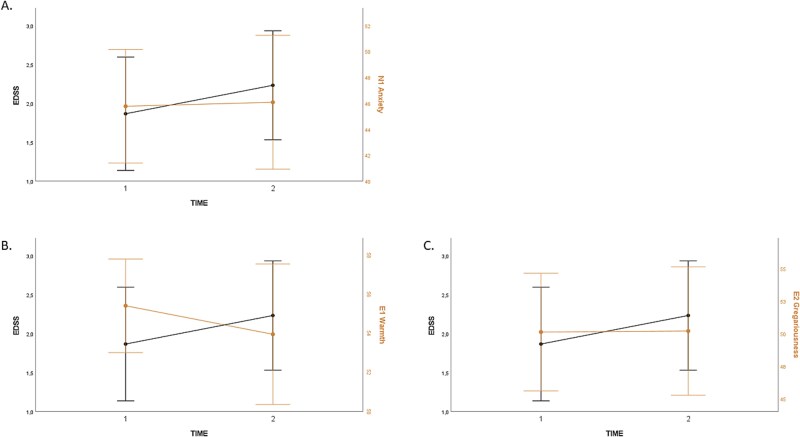
Longitudinal changes in EDSS as well as the personality traits of anxiety (A), warmth (B) and gregariousness (C). *Legends.* N1 = first facet of Neuroticism, E1 = first facet of Extraversion, E2 = second facet of Extraversion. Lines in the figures are interpolation lines.

Changes in EDSS, Anxiety, Warmth, and Gregariousness are represented in Fig.  2.

The PGEE analysis yielded results consistent with the classical GEE models, supporting the robustness of our findings (see Figs S4–S6).

## DISCUSSION

In this study, we explored the longitudinal relationships between personality traits and disability progression in PwMS. Our findings were congruent with our hypotheses and revealed significant associations between certain personality traits and the trajectory of disability over time. This supports the hypothesis that traits such as Neuroticism and Extraversion may be associated with disability progression. These two personality factors are frequently associated with important problems present in MS, such as mood and cognitive difficulties, as well as fatigue (Chu et al., 2022; Ciancio et al., 2023; Roy et al., 2018b).

The facet-level analysis revealed that Anxiety, Warmth, and Gregariousness might help in better understanding the associations between Neuroticism, Extraversion, and disability progression. These results suggest that an increase in disability is mainly associated with an increase in *the tendency to feel diffuse anxiety* (Anxiety characteristics) as well as a decrease in *intimacy in interpersonal relationships* (Warmth characteristics) and in *fondness for others’ company* (Gregariousness characteristics). This precision regarding which specific personality traits change alongside disability progression seems highly important to clarify the mechanisms that could explain these associations. Assuming that there are bidirectional influences between personality and disability in MS, the use of personality assessment could help identify more vulnerable individuals. Given the characteristics of the three facets whose changes were associated with an increase in disability, an important aspect to consider is the impact these changes could have on social participation. Past studies have shown that Neuroticism and Extraversion were closely related to health-related quality of life in MS, including social functioning (Zarbo et al., 2016). We believe that early detection of such changes could help improve clinical management by enabling a more person-centered approach that takes individual differences into account. Identifying these changes may also help prevent potential social isolation, which might contribute to increased disability. Future studies should consider this possible social impact and test whether personality changes are more closely associated with disability in specific life domains.

Previous research has shown that personality changes occur particularly in the traits of Extraversion and Conscientiousness (Fuchs et al., 2018). Contrary to these results, our mean group comparison did not show significant changes in the five factors of personality or the thirty facets, pointing to no observable trends at the group level (see Supplementary Material). However, our analysis also revealed considerable individual differences that were not captured in these classical mean group analyses (see Figs S1–S3). The lack of significant whole-group differences in our personality traits does not negate the value of our GEEs. While classical group comparisons treat personality changes as uniform across the sample, GEE accounts for intra-individual variability by evaluating changes over time within individuals and adjusting for correlations among repeated measures. This makes GEEs particularly well-suited for our data. Unlike classical group-level analyses, which assume homogeneous correlation structures across subjects, GEEs can accommodate variability in within-subject correlation, regardless of how the correlation structure is defined (Zeger & Liang, 1986). It is important to note that the individual variability observed may be partially driven by cognitive status, emotional distress, and mood-related aspects (Bruce & Lynch, 2011; Fuchs et al., 2018; Watson & Naragon-Gainey, 2014). Roy et al. 2018a also reported a high degree of heterogeneity, noting that individuals without cognitive deficits showed smaller changes in personality over time. Our findings mirror this heterogeneity and underscore the complex, multifactorial nature of personality change in MS.

A final point we wished to discuss is the question of causality between personality changes and disability progression. Indeed, we wanted to emphasize that this study is purely correlational. We did not postulate any clear causality; rather, we believe there is a dynamic relationship between personality changes and disability progression, with likely bidirectional influences. Similarly, we did not postulate any specific underlying mechanisms to explain personality changes in our group of PwMS. We believe these changes are multifactorial, involving aspects directly related to the disease mechanism (e.g., brain changes), indirectly related (e.g., adaptation to a diagnosis of a chronic disease, mood disorders, therapy etc.), as well as factors unrelated to MS, such as important life events or societal contexts—for example the COVID-19 pandemic, which has been linked to changes in personality (Rudolph & Zacher, 2023).

It is important to interpret our results in light of the study limitations, mainly the small sample size, which could limit the generalizability of our results. Additionally, the characteristics of the individuals who agreed to participate in the second evaluation, which included an exhaustive neuropsychological assessment, might have introduced a selection bias. As highlighted by our preliminary analysis, individuals who completed the longitudinal follow-up had a higher initial level of Openness. We believe it is important to acknowledge this, as it might have introduced a selection bias that could affect the generalizability of our results. Nonetheless, we wish to emphasize that this may represent a common bias in longitudinal research, as previous studies have shown that individuals who volunteer for follow-ups in research have different personality profiles than those who do not, notably higher levels of Openness (Marcus & Schütz, 2005). Also, some relevant aspects that might impact personality and/or the associations between personality and disability were not included in this study. Notably, treatment choice and adherence, depressive and anxious symptoms, fatigue, and chronic pain. Future larger studies should consider integrating these aspects as potential mediators of the association between personality and disability. It could also be interesting to break down MS subtypes to investigate whether these associations vary across subtypes. Finally, we believe that to better understand the role of personality in disability progression, it is important to include a more holistic measure of disability that captures daily life functioning. In this context, the use of the WHODAS 2.0, which assesses disability in five life domains—Cognition, Mobility, Self-Care, Getting Along, Life Activities, and Participation—(Üstün, 2010), should be preferred to assess disability, especially as it has been validated in MS (Young et al., 2023).

## CONCLUSION

Our study extends the literature by highlighting the importance of considering personality and its associations with disability in MS from a longitudinal perspective. Our results suggest that, while classical group comparisons may overlook key insights, more granular approaches like GEEs provide a more accurate understanding of how personality traits and disability progression are interrelated. Further research should explore how additional factors—including mood disturbances and cognitive difficulties – interact with personality traits in MS, as this may help inform clinical practice in managing the disease’s multifactorial nature.

## Supplementary Material

Supplementary_Material_(1)_acaf063

Data_SI_acaf063

## Data Availability

The data used for the analysis are provided as Supplementary Materials.
