# Supplementary material for: Linking Personality Traits to Disability Progression in Multiple Sclerosis: A Longitudinal Analysis
Source: Arch Clin Neuropsychol. 2025 Jul 15;40(8):1526–32. doi: 10.1093/arclin/acaf063 (PMC12644050; doi:10.1093/arclin/acaf063)
Supplement: Supplementary_Material_(1)_acaf063 [file supplementary_material_(1)_acaf063.docx]

**Supplementary Material**

*S1 - Stability of personality traits at the group level*

Regarding personality, the paired sample tests failed to highlight any significant change at the group level for the five factors and the thirty facets (see Table S1). However, observable individual differences in personality changes were evident (see Figure S1), highlighting variability in how personality traits evolved over time. These individual differences warrant further exploration, particularly in relation to disability progression and other potential moderating or mediating factors.

**Table S1.** Comparison of levels on the five personality traits as well as their thirty facets at the two time points

|  |  |  |  | **Statistics t or w^#^** |  | **ddl** |  | **p** |  |
| --- | --- | --- | --- | --- | --- | --- | --- | --- | --- |
| Neuroticism |  |  |  | 0.1146 |  | 27 |  | 0.910 |  |
| Extraversion |  |  |  | -0.4628 |  | 27 |  | 0.647 |  |
| Openness |  |  |  | 0.3192 |  | 27 |  | 0.752 |  |
| Agreeableness |  |  |  | -0.3389 |  | 27 |  | 0.737 |  |
| Conscientiousness |  |  |  | -0.5343 |  | 27 |  | 0.597 |  |
| N1 Anxiety |  |  |  | 0.1040 |  | 27 |  | 0.918 |  |
| N2 Angry Hostility |  |  |  | -0.9580 |  | 27 |  | 0.347 |  |
| N3 Depression |  |  |  | 0.9690 |  | 27 |  | 0.341 |  |
| N4 Self-Consciousness |  |  |  | -0.1319 |  | 27 |  | 0.896 |  |
| N5 Impulsiveness |  |  |  | 1.0004 |  | 27 |  | 0.326 |  |
| N6 Vulnerability |  |  |  | 0.0000 |  | 27 |  | 1.000 |  |
| E1 Warmth |  |  |  | 153.0**^#^** |  | - |  | 0.943 |  |
| E2 Gregariousness |  |  |  | -0.4002 |  | 27 |  | 0.692 |  |
| E3 Assertiveness |  |  |  | 0.0263 |  | 27 |  | 0.979 |  |
| E4 Activity |  |  |  | -0.0185 |  | 27 |  | 0.985 |  |
| E5 Excitement Seeking |  |  |  | -0.6772 |  | 27 |  | 0.504 |  |
| E6 Positive Emotions |  |  |  | 143.5**^#^** |  | - |  | 0.864 |  |
| O1 Fantasy |  |  |  | -0.5896 |  | 27 |  | 0.560 |  |
| O2 Aesthetics |  |  |  | 0.7063 |  | 27 |  | 0.486 |  |
| O3 Feelings |  |  |  | 1.3303 |  | 27 |  | 0.195 |  |
| O4 Actions |  |  |  | 0.6442 |  | 27 |  | 0.525 |  |
| O5 Ideas |  |  |  | 0.5599 |  | 27 |  | 0.580 |  |
| O6 Values |  |  |  | -0.8564 |  | 27 |  | 0.399 |  |
| A1 Trust |  |  |  | 0.2323 |  | 27 |  | 0.818 |  |
| A2 Straightforwardness |  |  |  | 170.0**^#^** |  | - |  | 0.576 |  |
| A3 Altruism |  |  |  | -0.7502 |  | 27 |  | 0.460 |  |
| A4 Compliance |  |  |  | 164.5**^#^** |  | - |  | 0.563 |  |
| A5 Modesty |  |  |  | 138.0**^#^** |  | - |  | 0.517 |  |
| A6 Tender Mindedness |  |  |  | 194.5**^#^** |  | - |  | 0.088 |  |
| C1 Competence |  |  |  | 0.5992 |  | 27 |  | 0.554 |  |
| C2 Order |  |  |  | 0.1119 |  | 27 |  | 0.912 |  |
| C3 Dutifulness |  |  |  | 88.5**^#^** |  | - |  | 0.221 |  |
| C4 Achievement Striving |  |  |  | -0.0304 |  | 27 |  | 0.976 |  |
| C5 Self-Discipline |  |  |  | 0.7076 |  | 27 |  | 0.485 |  |
| C6 Deliberation |  |  |  | -1.3063 |  | 27 |  | 0.202 |  |

**Table S2.** Neuropsychological domains and functions assessed to describe the cognitive performance of the individuals included in the study

| Domain | Functions | Name of tests |
| --- | --- | --- |
| Long term memory | Episodic verbal | French version of Grober and Buschke free/cued recall (RL/RI 16) paradigm (Van der Linden et al., 2004) |
|  | Episodic visuospatial | Subtest “Designs I and II” of the Wechsler Memory Scale – Fourth Edition (Wechsler, 2009) |
| Attention | Phasic alertness | Test for Attention Performance (Zimmermann & Fimm, 2007) |
|  | Divided attention |  |
|  | Sustained attention |  |
| Executive functions | Inhibition | Stroop task from GREFEX battery (Roussel & Godefroy, 2008) |
|  | Mental flexibility | Trail Making Test from GREFEX battery (Roussel & Godefroy, 2008) |
|  | Verbal working memory | Backward Digit Span from WAIS-IV (Wechsler, 2008) |
|  | Visuo-spatial working memory | Backward Corsi test from WMS-III (Wechsler, 1997) |
| Language | Semantic image and word matching | Cognitive Language Assessment Battery - Batterie d’évaluation cognitive du langage (BECLA, Macoir et al., 2016) |
|  | Naming |  |
|  | Repetition |  |
| Visual perception | Object perception – Incomplete letters | Visual Object and Space Perception battery (Warrington & James, 1991) |
|  | Spatial perception – Number location |  |
| Ideomotor praxis |  | Moroni praxis battery (Warrington & James, 1991) |

**Table S3.** Distribution of cognitive performance across six cognitive domains

| **Domain** | **Percentage of score <2th percentile** | **Percentage of score >2th - <5th percentile** | **Percentage of score > 5th - <16th percentile** | **Percentage of score >16th - <84th percentile** | **Percentage of score >84th - <95th percentile** | **Percentage of score >95th - <98th percentile** | **Percentage of score >98th percentile** |
| --- | --- | --- | --- | --- | --- | --- | --- |
| Long term memory | 0% | 14.28% | 28.57% | 100% | 75% | 60.71% | 25% |
| Attention | 7.41% | 14.81% | 59.25% | 100% | 81.48% | 48.15% | 40.74% |
| Executive functions | 17.86% | 0% | 35.71% | 100% | 32.14% | 21.43% | 0% |
| Language | 10.71% | 14.29% | 32.14% | 100% | 0% | 10.71% | 0% |
| Visual perception | 3.57% | 0% | 14.29% | 100% |  |  |  |
| Ideomotor praxis | 0% | 0% | 7.41% | 100% |  |  |  |

*Notes.* Memory (15 sub-tests); Attention (15 sub-tests); Executive functions (6 sub-tests); Language (5 sub-tests); Visual perception (2 sub-tests); Ideomotor praxis (3 sub-tests). For visual perception and praxis as the maximum of points are expected there is no percentiles higher than 84.

**Table S4.** Comparison of clinical and personality variables between individuals who accepted the follow-up and those who did not

|  |  |  |  | **Statistics t, w^#^ or X^2*^** |  | **ddl** |  | **p** |  |
| --- | --- | --- | --- | --- | --- | --- | --- | --- | --- |
| Age |  |  |  | 206^#^ |  | - |  | 0.928 |  |
| Time since disease onset |  |  |  | 205.5^#^ |  | - |  | 0.918 |  |
| EDSS |  |  |  | 141^#^ |  | - |  | 0.142 |  |
| Sex |  |  |  | 0.84* |  | 1 |  | 0.359 |  |
| MS type |  |  |  | 0.21* |  | 1 |  | 0.646 |  |
| Neuroticism |  |  |  | 1.51 |  | 41 |  | 0.138 |  |
| Extraversion |  |  |  | -1.76 |  | 41 |  | 0.086 |  |
| Openness |  |  |  | -3.53 |  | 41 |  | 0.001 |  |
| Agreeableness |  |  |  | 156.5^#^ |  | - |  | 0.177 |  |
| Conscientiousness |  |  |  | 205 |  | - |  | 0.909 |  |


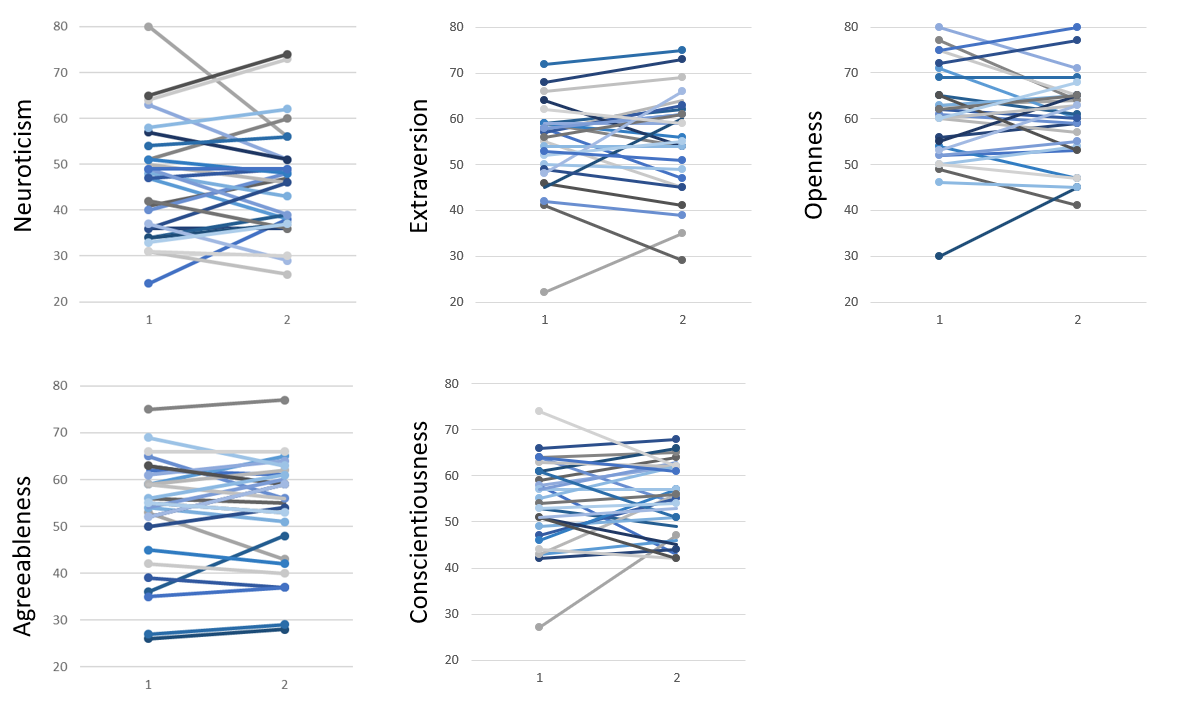


**Figure S1**. Representation of individual changes on the five personality traits described by the Five Factor Model in a 4-years interval. Legends. 1 = first time point, 2 = second time point


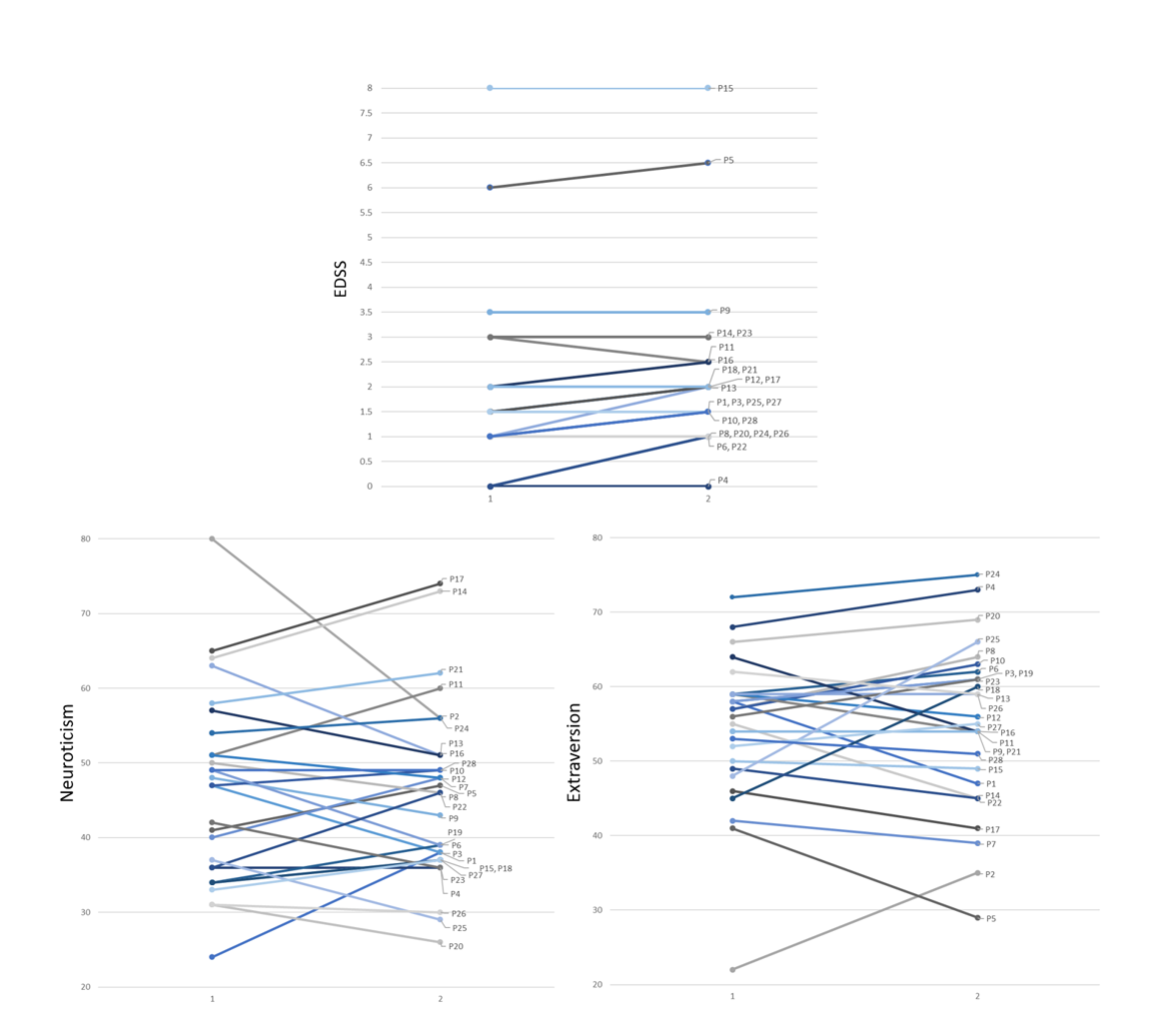


**Figure S2.** Visual representation of individual changes in EDSS, Neuroticism and Extraversion in a 4-years interval. Legends. 1 = first time point, 2 = second time point, EDSS = Expanded Disability Status Scale, P1 (…) = person number.


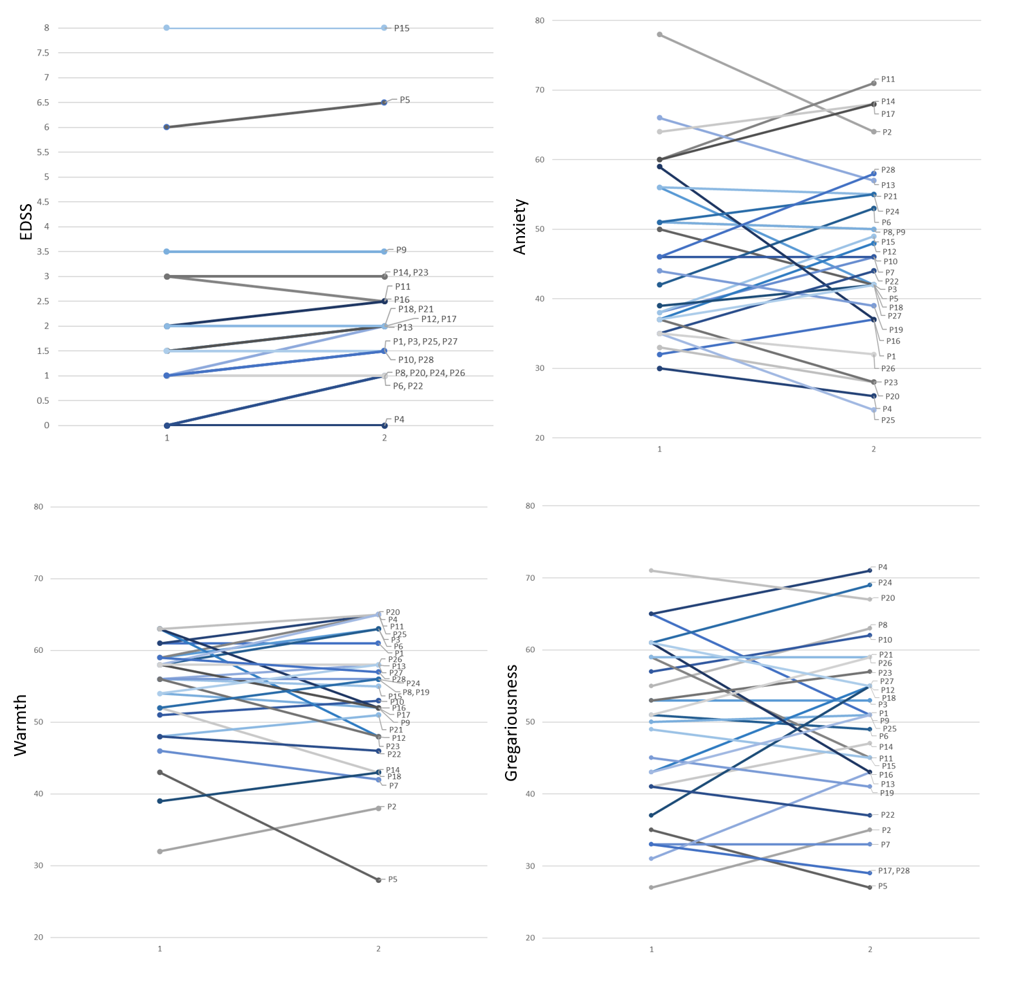


**Figure S3.** Visual representation of individual changes in EDSS, Anxiety, Warmth and Gregariousness in a 4-years interval. Legends. 1 = first time point, 2 = second time point, EDSS = Expanded Disability Status Scale, P1 (…) = person number.

**Figure S4.** Output of the Penalized Generalized Estimating Equations for the model with the five personality factors. Legends. DM = time since disease onset, N = Neuroticism, E = Extraversion, O = Openness, C = Conscientiousness

**Figure S5.** Output of the Penalized Generalized Estimating Equations for the model with the six facets of Neuroticism. Legends. DM = time since disease onset, N1 = Anxiety, N3 = Depression, N4 = Self-Consciousness, N5 = Impulsiveness, N6 = Vulnerability

**Figure S6.** Output of the Penalized Generalized Estimating Equations for the model with the six facets of Extraversion. Legends. DM = time since disease onset, E1 = Warmth, E2 = Gregariousness, E3 = Assertiveness, E5 = Excitement-Seeking, E6 = Positive Emotions

**References**

Macoir, J., Gauthier, C., Jean, C., & Potvin, O. (2016). BECLA, a new assessment battery for acquired deficits of language: Normative data from Quebec-French healthy younger and older adults. *Journal of the Neurological Sciences*, *361*, 220-228.

Roussel, M., & Godefroy, O. (2008). La batterie GREFEX: données normatives. Fonctions exécutives et pathologies neurologiques et psychiatriques., 231-252.

Van der Linden, M., Coyette, F., Poitrenaud, J., Kalafat, M., Calicis, F., Wyns, C., & Adam, S. (2004). Ii. L’épreuve de rappel libre/rappel indicé à 16 items (RL/RI-16). *L’Evaluation des troubles de la mémoire. Solal*, 25-47.

Warrington, E., & James, M. (1991). The Visual Object and Space Perception Battery, Thames Valley Test Company, Bury St. *Edmunds, UK*.

Wechsler, D. (1997). The Wechsler Memory Scale, San Antonio, Tex, Psychological Corp. *Published online*.

Wechsler, D. (2008). *WAIS-IV : Wechsler Adult Intelligence Scale - Fourth Edition*. Pearson.

Wechsler, D. (2009). *Wechsler memory scale: WMS-IV; technical and interpretive manual*. Pearson.

Zimmermann, P., & Fimm, B. (2007). *Test for attentional performance (TAP), Version 2.1, operating manual.* . PsyTest.
